# Transplantable Murine Tumors in the Studies of Peptide Antitumor Vaccines

**DOI:** 10.3389/or.2023.12189

**Published:** 2024-01-08

**Authors:** Aleksandr V. Ponomarev, Irina Zh. Shubina, Zinaida A. Sokolova, Maria A. Baryshnikova, Vyacheslav S. Kosorukov

**Affiliations:** N. N. Blokhin National Medical Research Center of Oncology, Moscow, Russia

**Keywords:** antitumor vaccines, peptides, antigens, neoantigens, transplantable murine tumors

## Abstract

Numerous studies have shown that antitumor vaccines based on synthetic peptides are safe and can induce both CD8^+^ and CD4^+^ tumor-specific T cell responses. However, clinical results are still scarce, and such approach to antitumor treatment has not gained a wide implication, yet. Recently, particular advances have been achieved due to tumor sequencing and the search for immunogenic neoantigens caused by mutations. One of the most important issues for peptide vaccines, along with the choice of optimal adjuvants and vaccination regimens, is the search for effective target antigens. Extensive studies of peptide vaccines, including those on murine models, are required to reveal the effective vaccine constructs. The review presents transplantable murine tumors with the detected peptides that showed antitumor efficacy as a vaccine compound.

## Introduction

Peptide-based antitumor vaccines include synthetic tumor-associated or tumor-specific peptides or combinations of peptides and are designed to activate peptide-specific T cells *in vivo*.

Peptide vaccines have a number of advantages over other types of vaccines, especially in terms of safety and simple manufacturing techniques. Unlike other antigen-specific therapies, such as CAR T cells, an important characteristic of peptide-based vaccines is that they can easily and economically combine several different antigens within a single injection. Thus, multipeptide vaccines may solve the problem of antigen loss and reduce the risk of tumor escape from the immune reactions, which often results from the received chemotherapy.

The main difficulties in creating peptide antitumor vaccines are the selection of the most optimal antigens and the search for effective but non-toxic adjuvants [1]. An ideal antigen for a tumor vaccine should be highly immunogenic and strongly expressed in all cancer cells (but not in normal cells), and the survival of cancer cells should depend on these antigens. Tumor antigens could be classified as tumor-associated antigens (TAA) and tumor-specific antigens. Tumor-associated antigens are also expressed in normal cells, though their expression in tumors is several times higher. TAA include differentiation, cancer-testis (CT) and overexpressed antigens. The limitation for TAA use in vaccines is potential elimination of the activated T-cells that recognize TAA due to the central immune tolerance of the thymus, which will eventually affect the vaccine efficacy. Moreover, TAAs are also expressed in non-malignant tissues, which increases the risk of vaccine-induced autoimmune toxicity. However, clinical studies of antitumor vaccines have demonstrated only rare autoimmune events [2–5].

Tumor-specific antigens include a number of antigens of viral origin and antigens emerging after mutations in cellular proteins (neoantigens). It is assumed that during tumor growth cancer cells accumulate a large number of somatic mutations which lead to the formation of neoantigens. Some of these neoantigens are highly immunogenic and may be regarded as target molecules for peptide vaccines. Neoantigens have original tumor specificity and therefore they are not affected by central or peripheral tolerance. A promising potential of neoantigen-based vaccines has been studied in preclinical and early clinical trials evaluating neoantigen-based peptide vaccines in a number of cancers [6–8]. Modern sequencing and bioinformatics technologies are now available for the development of personalized vaccines based on neoantigens. However, identification of immunogenic neoantigens is still a challenge and various bioinformatics tools are being designed to improve prediction and selection of candidate neoepitopes for antitumor vaccines. Besides, different tumors have a rather low mutational burden and selection of a necessary range of immunogenic neoantigens becomes a problem.

Preclinical studies of peptide vaccines involve various models of transplantable mouse tumors. Mouse models include a class of antigen-transfected tumors. For instance, mouse TC-1 tumor cells express E6 and E7 oncogenes derived from human papillomavirus (HPV-16). Thus, TC-1 cells may be considered as a mouse model of HPV-16-induced cervical cancer. Transfected cell lines have an expressed antigen as a target for an antitumor vaccine that can be used for studying new adjuvants.

The review presents the description of mouse tumor lines for studying new adjuvants or combination of new peptide antigens with the previously identified peptides in antitumor vaccines. The purpose of the review is to summarize the information about the cell lines of transplantable mouse tumors used in preclinical studies of peptide antitumor vaccines.

## Transplantable Mouse Tumor Models With the Available Immunogenic Peptides

The table below comprises a number of models of transplantable mouse tumors with the available immunogenic peptides (Table 1). The study reports describe antitumor functions of these peptides being the base for antitumor vaccines.

**TABLE 1:** Transplantable mouse tumor cell lines with the available peptides that have antitumor activity as a vaccine compound.

Cell line	Peptide	Amino-acid sequence (if indicated)	Type of antigen	References
EG7	OVA_257-264_	SIINFEKL	TF	[11]
ТС1	E7_49–57_	RAHYNIVTF	TF	[13]
	E7_43-77_	GQAEPDRAHYNIVTFCCKCDSTLRLCVQSTHVDIR	TF	[10, 13]
	SLP_HPV_	GQAEPDRAHYNIVTFCCKCDS	TF	[14]
TRAMP С1	PAP-114-128	MSAMTNLAALFPPEG	ТАА	[17]
В16-F10	TRP2	VYDFFVWL	TAA	[23]
	a) MUT44	–	neu	[24]
	b) MUT30			
	a) Gal3St1	a) AAPCSPIPNEPVAAT **A** ANGSAGGCQPR	neu	[26]
	b) M30	b) PSKPSFQEFVDWE **N** VSPELNSTDQPFL	neu	
	c) TRP2	c) VYDFFVWL	ТАА	
	M30	PSKPSFQEFVDWENVSPELNSTDQPFL	neu	[11]
4Т1	M25	KDYTAAGFSSFQKLRLDLTSMQIITTD	neu	[11]
	NeoAG 1, 2, 3, 4	A pool of 4 long peptides	neu	[30]
	a) CD151 1	a) IYKVEGGCI	ТАА	[31]
	b) CD151 2	b) DWQDSEWIRSG		
TNBC E0771.LMB	LP 1, 5, 9, 13 and 15	Two sets of peptides for immunization	neu	[35]
		a) LP 1, 5, 9		
		b) LP 13, 15		
Н22	a) CD151 1	a) IYKVEGGCI	ТАА	[31]
	b) CD151 2	b) DWQDSEWIRSG		
	neoantigen	HTDAHAQAFAALFDSMH	neu	[36]
Hep-55.1C	Peptides encoded 5, 7, 9, 12, 16, 18	A pool of 6 peptides	neu	[37]
Hepa1-6	–	WDTCTTYKWQKTLEGHD	neu	[39]
	a) Mapk3_S284F	a) MKARNYLQFLPSKTKVA	neu	[38]
	b) Lmf1_F523V	b) RGEHYRYKVSLPGGQHA		
	c) Samd91_K752M	c) HVLWDLKQMFRCAVLKN		
	d) Traf7_C403W	d) WDTCTTYKWQKTLEGHD		
	e) Dtnb_K40T	e) LSTYRTACTLRFVQKRC		
	f) Lbr_A341P	f) LYTHFLQLPLAATGFSV		
	g) Ptpn2_I383T	g) KRWLYWQPTLTKMGFVS		
МС-38	a) Adpgk	a) GIPVHLELASMTNMELMSSIVHQQVFPT	neu	[18]
	b) Reps1	b) GRVLELFRAAQLANDVVLQIMELCGATR		
	c) Dpagt1	c) EAGQSLVISASIIVFNLLELEGDYR		
	Rpl18	KAGGKILTFD **R** LALESPK	neu	[40]
СТ26	AH1	SPSYVYHQF		[42]
	a) ME1	a) LHSGQNHLKEMAISVLEARACAAAGQS	neu	[11]
	b) ME4	b) WKGGPVKIDPLALMQAIERYLVVRGYG		
Panc02	a) Meso_351-358_	–	ТАА	[45]
	b) Meso_612-619_			
	–	A pool of 12 long peptides	neu	[46]
YTN16	mCdt1	КTVYP **M** SYRF	neu	[47]
ASB-XIV	mPhf3	MGPQKFYQV	neu	[48]
LLC	a)Mut1	a) KPFYPALISYMGSGPVVAMVWEGP	neu	[49]
	b)Mut5	b) ELELALSPIHYSSAIPAAGSNQVT		
	L82	YDECGLAYVK[L]QGIRFQEKPY	neu	[51]
SCC VII	a) –	a) A pool of five 20-mer peptides	neu	[54]
	b) Cltc (Mut_48)	b) –		
MOC22	mICAM1	DQILETQRTLTVYNFSALVLTLSQLEVS	neu	[55]
CT2A	a) Epb4	a) ELEQFESTIGFKLPNLRAAKRLWK	neu	[56]
	b) Pomgnt1	b) ECIIPDVSLSYHFGIVGLNMNGYF		
	c) Plin2	c) QQLQTTCQTVLV NAQRLPQNIQDQA		
GL261	mImp3	–	neu	[56]
	a) Imp3	a) AALLNKLYA	neu	[58, 59]
	b) Ntrk1	b) MSLQFMTL		
	c) Rtn2	c) GAIFNGFTL		
SMA-560	Odc1	KQVSQIKYAASNGVLMMTFDSEIELMKVA	neu	[60]

Abbreviations: TF, transfected; TAA, tumor-associated antigen; neu, neoantigen.

### EG7

EG7 is a lymphoma cell line (derived from the EL4 line) that expresses ovalbumin and grows in mice C57BL/6. The line is often used to study new vaccine adjuvants, since it expresses a well-known antigen ovalbumin and antitumor vaccines and adjuvants usually show a pronounced effect towards EG7. M. Matsumoto et al. studied their original adjuvant in the EG7 model in the therapeutic mode with ovalbumin as an antigen. The study results showed a significant reduction of the tumor size [9]. Other authors, R. Heidenreich et al., also studied a new adjuvant as a vaccine component with the antigen ovalbumin in prophylactic and therapeutic modes. It should be noted that the researchers registered moderate inhibition of the tumor growth in the prophylactic mode, and after vaccination the tumors did not express ovalbumin. Probably, immune surveillance could have led to the selection of OVA-negative tumor cells [10]. Zh. Jing et al. reported a study of a vaccine construct containing OVA257-264 peptide that could significantly inhibit the EG7 tumor growth in both prophylactic and therapeutic regimens [11].

### TC-1

The TC-1 cell line was derived from the primary lung epithelial cells of the C57BL/6 mice. They are transformed by the c-Ha-ras oncogene and consistently express E6 and E7 oncogenes derived from the human papillomavirus (HPV-16). These cells provide progressive tumor growth after infection with the oncogenic dose [12]. TC-1 cells might be regarded as a mouse model of HPV-16-induced cervical cancer. A study [13] showed that the therapeutic vaccination with long peptide E7_43-77_ with an adjuvant had a significantly stronger antitumor effect than that with short peptide E7_49-57_ and an adjuvant. Another study showed a very significant antitumor efficacy of long peptide E7_43-77_ and an adjuvant used in the prophylactic and therapeutic regimens [10]. G.G. Zom et al. immunized the mice with transplantable TC-1 tumors by the vaccines obtained by conjugating an adjuvant with the SLP_HPV_ peptide or by plain mixing an adjuvant and the SLP_HPV_ peptide. The results showed that the conjugation, but not the mixture, of the adjuvant and SLP_HPV_ significantly increased the survival of mice [14]. Other researchers, E.K. Duperret et al. performed a study to search for neoantigens in the tumor. The authors found that a DNA vaccine based on the detected neoantigens inhibited the tumor growth [15].

### TRAMP C1

TRAMP C1 is a transgenic prostate adenocarcinoma of the C57BL/6 mice [16]. Patients with human prostate cancer have an overexpressed prostatic acid phosphatase (PAP), which is a tumor-associated antigen. Regarding an antitumor vaccine, it is important to note that PAP sequences have a high degree of homology between murine and human proteins. A vaccine with the PAP-114-128 peptide inhibited the growth of the TRAMP C1 mouse tumor in both prophylactic and therapeutic regimens [17]. Nevertheless, an attempt to find immunogenic tumor mutations by mass spectrometry and exome sequencing showed a small number of mutations in that cell line and no immunogenic neoantigens were found [18].

### ID8

ID8 cell line is used as a syngeneic mouse model for ovarian cancer. The cell line was derived from the epithelial cells of the surface epithelium of the ovaries of C57BL/6 mice. S.D. Martin et al. searched for neoantigens in the ID8 cells and found just a small number of mutations. The authors used peptide vaccines based on the detected neoantigens, but the vaccination could not inhibit the progression of the tumor in either prophylactic or therapeutic regimens [19]. E.K. Duperret et al. vaccinated the animals with a prophylactic neoantigen-based DNA vaccine and achieved an increased survival of the mice [15].

### B16-F10

B16-F10 is a metastatic clone of the B16 cell line derived from spontaneous melanoma of C57BL/6 mice. B16-F10 cells have low immunogenicity, and vaccination with irradiated tumor cells could not protect the mice from tumor growth after transplantation of live B16-F10 cells [20, 21], which might be partly explained by low expression of MHC class I and II molecules [22]. M.B. Bloom et al. have found that tyrosinase-related protein 2 (TRP2) is a tumor rejection antigen for melanoma B16-F10. Their studies showed that cytotoxic lymphocytes stimulated *in vitro* with the TRP2 peptide could recognize B16-F10 tumor and had a therapeutic effect against established pulmonary metastases [23]. J.C. Castle et al. sequenced melanoma B16-F10 and found an antitumor effect associated with the two tested neoantigenic peptides MUT44 and MUT30. Each peptide included into a vaccine with an adjuvant was used in a therapeutic regimen and thus showed a decreased tumor growth compared with the control [24]. Another study showed an antitumor effect of an RNA vaccine including peptide sequence M30 [25]. Zh. Jing et al. used a vaccine construct with M30 peptide antigen in a prophylactic mode. The authors reported that the vaccine significantly inhibited the development of B16-F10 metastases in the mouse lungs [11]. In 2021 H. Lam et al. reported a study of the whole exome sequencing of melanoma B16-F10. It revealed another neoantigenic peptide Gal3St1, which was used in vaccination resulting in a moderate antitumor effect. On the other hand, vaccination with a set of peptides Gal3St1+M30+TRP2 with an adjuvant in the therapeutic mode led to a very strong antitumor effect. Studies on the B16-F10 model helped the authors to discover a phenomenon that some neoantigenic peptides may inhibit the immune responses. Immunization with such peptides led to accelerated tumor growth and canceled the effect of the protective vaccine [26]. Y. Zhang et al. proposed an original way to increase the immunogenicity of neoantigens. The authors modified 4 weak neoantigenic peptides B16-F10 and significant inhibition of tumor growth was observed in preventive and therapeutic regimens after immunization with this construct [27].

### 4T1

4T1 mammary carcinoma of BALB/C mice is highly tumorigenic and invasive and may have spontaneous metastases [28]. An RNA vaccine including peptide sequence M25 showed an essential antitumor effect [25]. Zh. Jing et al. immunized the mice with a vaccine construct including peptide M25 as an antigen in the prophylactic mode and noted a slight slowdown of the tumor growth [11]. M. Peterson et al. searched for the neoantigens that appeared due to the errors in the production of RNA resulting from the frameshift, but not due to the DNA mutations. The researchers tested the vaccines that included about 10 neoantigenic peptides with an adjuvant. The immunization was performed in the therapeutic regimen and the results showed a reduction in the incidence and rate of tumor growth, though it should be noted that the peptides were not studied individually [29]. M.O. Mohsen et al. performed a search for immunogenic neoantigens and selected 4 peptides for a more thorough study. Therapeutic immunization of mice with a vaccine construct including long peptides of these neoantigens led to inhibition of tumor growth [30]. W. Lin et al. completed a proteomic and bioinformatics analysis and identified protein CD151 as a potential tumor-associated antigen for a vaccine to be tested in the 4T1 and H22 cell lines. The preventive immunization with two peptides of protein CD151 with an adjuvant showed a lower rate of metastasis and fewer tumor nodes in the lungs of mice as compared with the control. Mice immunized with the peptide had a higher survival rate, as well [31]. C. Lhuillier et al. searched for potentially immunogenic neoantigens for this cell line and found only a small number of candidate genes. However, the expression of some candidate genes increased significantly after irradiation of 4T1 cells. The authors made a vaccine from an adjuvant and three peptides to those neoantigens (DHX58, CAND1 and ADGRF5-II). The antitumor effect was observed only when vaccination was combined with radiation therapy, but not when vaccination was used in mono-regimen [32]. L. Li et al. used sequencing and bioinformatics analysis to search for neoantigens for 4T1 cells and another murine mammary tumor E0771, though the mice were immunized with a DNA vaccine, but not with peptides [33].

### TNBC E0771.LMB

Metastatic tumor EO771.LMB was derived from poorly metastatic mammary tumor cells EO771 of C57BL/6 mice [34]. R. Brito Baleiro et al. performed whole exome sequencing and bioinformatics analysis to search for immunogenic neoantigens of the TNBC E0771.LMB cell line. Then the authors immunized the mice with a pool of long peptides, an adjuvant and a PD-1 inhibitor. The experimental animals had slower tumor growth and longer survival compared to the untreated control mice or animal group receiving the adjuvant and a PD-1 inhibitor [35]. Other researchers, such as L. Li et al. searched for and studied neoantigens of the E0771 cells [33].

### H22

H22 is a BALB/C mouse hepatocellular carcinoma cell line. As mentioned above, W. Lin et al. identified protein CD151 as a promising tumor-associated antigen for 4T1 and H22 murine transplantable tumors. When mice were vaccinated prophylactically with two CD151 peptides and an adjuvant, tumor growth significantly slowed down as compared to the control group [31]. D. Zhang et al. identified a neoantigen of the H22 cells. Prophylactic immunization with an adjuvant and the neoantigenic peptide inhibited the tumor growth. The authors designed a vaccine that prevented tumor growth in a part of experimental animals. Therapeutic vaccination with this construct led to slower tumor growth and improved survival [36].

### Hep-55.1C

Hep-55.1C is a hepatocellular carcinoma cell line derived from C57BL/6 mice. S.F. Yang et al. revealed neoantigens in cell lines Hep-55.1C and Dt81 Hepa1-6 by comparing their whole exome sequences with those of normal mouse liver. Mice were vaccinated with an adjuvant and a mixture of six neoantigenic peptides. Treatment of mice with the vaccine in a therapeutic regimen resulted in slower tumor growth and improved survival compared to administration of an adjuvant alone. Combining vaccination with anti-PD-1 therapy resulted in more significant tumor regression as compared to that after monotherapy [37].

### Hepa1-6

Hepa1-6 is a hepatoma cell line derived from C57L mice. H. Chen et al. performed tumor cell sequencing, bioinformatics analysis, and neoantigen screening and selected 7 peptides for immunization of mice. Therapeutic vaccination with a mixture of these peptides and the adjuvant Poly(I:C) (but not other adjuvants) led to the disappearance of tumors in 60% of mice. Immunization with each peptide separately showed lower antitumor efficacy [38]. Q. Zhao et al. included one neoantigenic peptide, specific to these tumor cells, into the vaccine construct and achieved a marked antitumor effect [39]. S.F. Yang et al. identified specific neoantigens for line Dt81 Hepa1-6 [37].

### MC-38

MC-38 is a cell line of the colon adenocarcinoma of C57BL/6 mice. The line is a tumor model with a high mutational burden. Vaccination with three predicted neoantigenic peptides together with an adjuvant generated effective T-cell immunity for significant tumor inhibition in prophylactic and therapeutic regimens [18]. B.J. Hos et al. revealed another immunogenic neoantigen Rpl18. Mice vaccinated with this peptide in a therapeutic regimen had a significantly lower tumor size compared to the control animals that received only an adjuvant [40]. B. Schrors et al. showed that MC38 colorectal adenocarcinoma cell lines derived from two different sources had significant differences in transcriptome and mutanome expression. There were also differences in the previously described immunogenic neoantigens [41].

### CT26

CT26 is a cell line of the colon carcinoma of BALB/C mice. The results of a study of a therapeutic RNA vaccine including a mixture of five neoantigenic peptides (pentatope 2) demonstrated a significant antitumor effect [25]. J.E. Slansky et al. showed that immunization with the AH1 (SPSYVYHOF) peptide, which is an epitope of the gp70 protein (a protein of the endogenous mouse virus) within a therapeutic dendritic cell vaccine resulted in the inhibition of tumor growth and an increase of the survival rate of mice [42]. Zh. Jing et al. designed a vaccine construct including 2 neoantigenic peptides ME1 and ME4. The researchers noted an inhibition of tumor growth after preventive and therapeutic vaccination. Besides, the therapeutic regimen showed improved survival of the experimental animals [11]. S. Feola et al. studied a vaccine construct consisting of an adenovirus coated with tumor antigen peptides that were similar to pathogen antigens due to molecular mimicry. The authors demonstrated the antitumor effectiveness of the designed vaccine. The peptide had a poly-K amino acid sequence at one end [43].

### Panc02

The Panc02 cell line is a C57BL/6 mouse model of pancreatic adenocarcinoma. A study found that Panc02 cells and human tumor samples overexpressed mesothelin [44]. I.C. Leao et al. used a prophylactic dendritic cell vaccine, where the antigen included two peptides to mesothelin Meso_351-358_ and Meso_612-619_. The vaccination increased the survival rate of the mice [45]. H.L. Kinkead et al. performed the whole exome sequencing, RNA sequencing (RNASeq), and the NetMHC immunogenicity prediction algorithm to develop a neoantigenic Panc02 tumor vaccine. Therapeutic immunization with an adjuvant and 12 long peptides resulted in delayed tumor growth and increased survival of the mice. However, the researchers did not study individual peptides [46].

### YTN16

YTN16 is a transplantable cell line from gastric cancer of C57BL/6 mice. K. Nagaoka et al. identified the immunogenic neoantigenic peptide mCdt1 in these tumor cells. A therapeutic vaccine based on dendritic cells loaded with this peptide showed an effective inhibition of the YTN16 tumor growth [47].

### ASB-XIV

ASB-XIV is a BALB/c mouse lung carcinoma cell line. C. Sun et al. performed sequencing and bioinformatics analyses of the tumor and screening of neoepitope peptides, which revealed the immunogenic neoantigenic peptide mPhf3. Then the authors studied a preventive and therapeutic dendritic cell vaccine with the mPhf3 peptide and showed a significant inhibition of tumor growth compared to that of the control mice [48].

### LLC

Lewis lung carcinoma (LLC) is a tumor cell line derived from spontaneous epidermoid carcinoma of the C57BL/6 mouse lungs. T. Chen et al. performed a search for immunogenic neoantigenic peptides of the tumor and showed that therapeutic vaccination with neoantigenic peptides Mut1 or Mut5 with an adjuvant resulted in delayed tumor growth and increased survival of the mice compared with the tumor growth and survival rate after the use of the adjuvant alone [49]. J. Sun et al. also searched for immunogenic neoantigens for LLC and found 10 neoantigens. An RNA vaccine targeting these neoantigens showed a reasonable antitumor effect [50]. C. Sun et al. conducted the whole exome and transcriptome sequencing of LLC from the ATCC collection to predict the neoantigen expressions. The authors evaluated neoantigenic peptide candidates and identified the most effective peptide L82. The experimental animals received a dendritic cell (DC) vaccine based on L82 and an additional immunomodulator CpG in a prophylactic mode, which resulted in a partial inhibition of the LLC1 tumor growth [51]. H. Qin et al. performed a search for immunogenic neoantigens in the LLC cells. The immunization was carried out using adoptive transfer of the RNA vaccine-treated DC-induced T cells [52].

### SCC VII

Squamous cell carcinoma VII (SCC VII) is a spontaneous tumor of C3H mice and it has similar characteristics to human head and neck squamous cell carcinoma (HNSCC) [53]. J.S. Dolina et al. performed a search for neoantigens in the cell line. Prophylactic immunization with a pool of five 20-mer peptides and an adjuvant led to inhibition of tumor growth. The authors studied the five peptides individually, as well. Only vaccination with Cltc peptide (Mut_48) showed a pronounced antitumor effect [54].

### MOC22

MOC22 is an oral cancer cell line derived from C57BL/6 mice. P. Zolkind et al. identified the mICAM1 neoantigen expressed by MOC22 cells using a cancer immunogenomics pipeline and ELISPOT immunogenicity testing. Prophylactic immunization by neoantigen peptide with an adjuvant resulted in the rejection of MOC22 tumors in 80% of mice [55].

### CT2A

CT2A is a glioma cell line established from C57BL/6 mice. С.J. Liu et al. performed whole-exome DNA sequencing and RNA sequencing to search for neoantigens in the CT2A cells. The authors selected 3 neoantigenic peptides: Epb4, Pomgnt1, and Plin2. Therapeutic vaccination with the three peptides in combination with anti-PD-L1 treatment led to increased survival compared to the vaccine alone or anti-PD-L1 monotherapy [56].

### GL261

GL261 cells present a syngeneic animal glioma model of C57BL/6 mice. T.M. Johannes et al. identified tumor-specific neoantigens in the GL261 cells on the base of the whole exome sequencing of DNA and RNA [57]. Another study showed that therapeutic vaccination of mice with the neoantigen peptide mImp3 and an adjuvant resulted in significantly improved survival compared with the adjuvant alone [56]. T. Su et al. studied a vaccine construct including 3 neoantigen peptides (Imp3, Ntrk1, Rtn2) combined with checkpoint inhibitors in a therapeutic regimen. The results showed a slight improvement of the survival in mice with GL261 tumors [58]. Previously, L. Scheetz et al. demonstrated the antitumor efficacy of a vaccine construct with these three peptides in combination with anti-PD-L1 therapy [59].

### SMA-560

Cell line SMA-560 was established from glioma of VM/Dk mice. T.M. Johannes et al. searched for neoantigens in the SMA-560 murine tumor model and, using tetramers, found T cells specific to the Odc1 neoantigen [57]. A.M. Swartz et al. showed that therapeutic vaccination of mice with a long peptide of neoantigen Odc1 inhibited tumor growth [60].

## Discussion

Nowadays, a potential for antitumor peptide vaccination is widely studied. New algorithms of searching for effective antigens are being developed, and new adjuvants are being studied. The presented information on transplantable mouse tumors with the identified peptides could be helpful for preclinical studies of antitumor peptide vaccines. Transplantable tumor models provide an important basis for designing new methods for identification of immunogenic antigens and direct testing the antigen antitumor effectiveness *in vivo*. In particular, that refers to the neoantigens arising due to mutations. In addition, these models could be used for comparison of various approaches to vaccination, such as comparing different lengths of peptides, different adjuvants, etc. When the problems in antigen identification and adjuvant selection are solved, the potential for this method could add to clinical practice as an independent therapy or in combination with other methods. Combination with therapies that reduce immunosuppression of the tumor microenvironment, such as checkpoint inhibitors, could be especially effective.

## References

[1] Nelde A Rammensee HG Walz JS. The Peptide Vaccine of the Future. Mol Cell Proteomics (2021) 20:100022. 10.1074/mcp.R120.002309 33583769 PMC7950068

[2] Amos SM Duong CP Westwood JA Ritchie DS Junghans RP Darcy PK Autoimmunity Associated With Immunotherapy of Cancer. Blood (2011) 118(3):499–509. 10.1182/blood-2011-01-325266 21531979

[3] Walter S Weinschenk T Stenzl A Zdrojowy R Pluzanska A Szczylik C Multipeptide Immune Response to Cancer Vaccine IMA901 After Single-Dose Cyclophosphamide Associates With Longer Patient Survival. Nat Med (2012) 18(8):1254–61. 10.1038/nm.2883 22842478

[4] Rini BI Stenzl A Zdrojowy R Kogan M Shkolnik M Oudard S IMA901, a Multipeptide Cancer Vaccine, Plus Sunitinib Versus Sunitinib Alone, as First-Line Therapy for Advanced or Metastatic Renal Cell Carcinoma (IMPRINT): A Multicentre, Open-Label, Randomised, Controlled, Phase 3 Trial. Lancet Oncol (2016) 17(11):1599–611. 10.1016/S1470-2045(16)30408-9 27720136

[5] Feyerabend S Stevanovic S Gouttefangeas C Wernet D Hennenlotter J Bedke J Novel Multi-Peptide Vaccination in Hla-A2+ Hormone Sensitive Patients With Biochemical Relapse of Prostate Cancer. The Prostate (2009) 69(9):917–27. 10.1002/pros.20941 19267352

[6] Li L Goedegebuure SP Gillanders WE. Preclinical and Clinical Development of Neoantigen Vaccines. Ann Oncol (2017) 28(12):xii11–xii17. 10.1093/annonc/mdx681 29253113 PMC5834106

[7] Blass E Ott PA. Advances in the Development of Personalized Neoantigen-Based Therapeutic Cancer Vaccines. Nat Rev Clin Oncol (2021) 18(4):215–29. 10.1038/s41571-020-00460-2 33473220 PMC7816749

[8] Lybaert L Thielemans K Feldman SA van der Burg SH Bogaert C Ott PA. Neoantigen-Directed Therapeutics in the Clinic: Where Are We? Trends Cancer (2023) 9(6):503–19. 10.1016/j.trecan.2023.02.004 37055237 PMC10414146

[9] Matsumoto M Tatematsu M Nishikawa F Azuma M Ishii N Morii-Sakai A Defined TLR3-Specific Adjuvant That Induces NK and CTL Activation Without Significant Cytokine Production *In Vivo* . Nat Commun (2015) 6:6280. 10.1038/ncomms7280 25692975

[10] Heidenreich R Jasny E Kowalczyk A Lutz J Probst J Baumhof P A Novel RNA-Based Adjuvant Combines Strong Immunostimulatory Capacities With a Favorable Safety Profile. Int J Cancer (2015) 137(2):372–84. 10.1002/ijc.29402 25530186

[11] Jing Z Wang S Xu K Tang Q Li W Zheng W A Potent Micron Neoantigen Tumor Vaccine GP-Neoantigen Induces Robust Antitumor Activity in Multiple Tumor Models. Adv Sci (2022) 9(24):e2201496. 10.1002/advs.202201496 PMC9403634 35712770

[12] Lin KY Guarnieri FG Staveley-O'Carroll KF Levitsky HI August JT Pardoll DM Treatment of Established Tumors With a Novel Vaccine That Enhances Major Histocompatibility Class II Presentation of Tumor Antigen. Cancer Res (1996) 56(1):21–6. 8548765

[13] Zwaveling S Mota SCF Nouta J Johnson M Lipford GB Offringa R Established Human Papillomavirus Type 16-Expressing Tumors Are Effectively Eradicated Following Vaccination With Long Peptides. J Immunol (2002) 169(1):350–8. 10.4049/jimmunol.169.1.350 12077264

[14] Zom GG Willems MMJHP Khan S van der Sluis TC Kleinovink JW Camps MGM Novel TLR2-Binding Adjuvant Induces Enhanced T Cell Responses and Tumor Eradication. J ImmunoTherapy Cancer (2018) 6(1):146. 10.1186/s40425-018-0455-2 PMC6292168 30541631

[15] Duperret EK Perales-Puchalt A Stoltz RGHH Gh H Mandloi N Barlow J A Synthetic DNA, Multi-Neoantigen Vaccine Drives Predominately MHC Class I CD8+ T-Cell Responses, Impacting Tumor Challenge. Cancer Immunol Res (2019) 7(2):174–82. 10.1158/2326-6066.CIR-18-0283 30679156 PMC6622455

[16] Foster BA Gingrich JR Kwon ED Madias C Greenberg NM. Characterization of Prostatic Epithelial Cell Lines Derived From Transgenic Adenocarcinoma of the Mouse Prostate (TRAMP) Model. Cancer Res (1997) 57(16):3325–30. 9269988

[17] Saif JM Vadakekolathu J Rane SS McDonald D Ahmad M Mathieu M Novel Prostate Acid Phosphatase-Based Peptide Vaccination Strategy Induces Antigen-Specific T-Cell Responses and Limits Tumour Growth in Mice. Eur J Immunol (2014) 44(4):994–1004. 10.1002/eji.201343863 24338683

[18] Yadav M Jhunjhunwala S Phung QT Lupardus P Tanguay J Bumbaca S Predicting Immunogenic Tumour Mutations by Combining Mass Spectrometry and Exome Sequencing. Nature (2014) 515(7528):572–6. 10.1038/nature14001 25428506

[19] Martin SD Brown SD Wick DA Nielsen JS Kroeger DR Twumasi-Boateng K Low Mutation Burden in Ovarian Cancer May Limit the Utility of Neoantigen-Targeted Vaccines. PLoS One (2016) 11(5):e0155189. 10.1371/journal.pone.0155189 27192170 PMC4871527

[20] Dranoff G Jaffee E Lazenby A Golumbek P Levitsky H Brose K Vaccination With Irradiated Tumor Cells Engineered to Secrete Murine Granulocyte-Macrophage Colony-Stimulating Factor Stimulates Potent, Specific, and Long-Lasting Anti-Tumor Immunity. Proc Natl Acad Sci U S A (1993) 90(8):3539–43. 10.1073/pnas.90.8.3539 8097319 PMC46336

[21] Overwijk WW Restifo NP. B16 as a Mouse Model for Human Melanoma. Curr Protoc Immunol (2001) Chapter 20:Unit 20.1. 10.1002/0471142735.im2001s39 PMC2763508 18432774

[22] Seliger B Wollscheid U Momburg F Blankenstein T Huber C. Characterization of the Major Histocompatibility Complex Class I Deficiencies in B16 Melanoma Cells. Cancer Res (2001) 61(3):1095–9. 11221838

[23] Bloom MB Perry-Lalley D Robbins PF Li Y el-Gamil M Rosenberg SA Identification of Tyrosinase-Related Protein 2 as a Tumor Rejection Antigen for the B16 Melanoma. J Exp Med (1997) 185(3):453–60. 10.1084/jem.185.3.453 9053445 PMC2196033

[24] Castle JC Kreiter S Diekmann J Lower M van de Roemer N de Graaf J Exploiting the Mutanome for Tumor Vaccination. Cancer Res (2012) 72(5):1081–91. 10.1158/0008-5472.CAN-11-3722 22237626

[25] Kreiter S Vormehr M van de Roemer N Diken M Lower M Diekmann J Mutant MHC Class II Epitopes Drive Therapeutic Immune Responses to Cancer. Nature (2015) 520(7549):692–6. 10.1038/nature14426 25901682 PMC4838069

[26] Lam H McNeil LK Starobinets H DeVault VL Cohen RB Twardowski P An Empirical Antigen Selection Method Identifies Neoantigens That Either Elicit Broad Antitumor T-Cell Responses or Drive Tumor Growth. Cancer Discov (2021) 11(3):696–713. 10.1158/2159-8290.CD-20-0377 33504579

[27] Zhang Y Lin Z Wan Y Cai H Deng L Li R. The Immunogenicity and Anti-Tumor Efficacy of a Rationally Designed Neoantigen Vaccine for B16F10 Mouse Melanoma. Front Immunol (2019) 10:2472. 10.3389/fimmu.2019.02472 31749795 PMC6848027

[28] Pulaski BA Ostrand-Rosenberg S. Mouse 4T1 Breast Tumor Model. Curr Protoc Immunol (2001) Chapter 20:Unit 20.2. 10.1002/0471142735.im2002s39 18432775

[29] Peterson M Murphy SN Lainson J Zhang J Shen L Diehnelt CW Comparison of Personal and Shared Frameshift Neoantigen Vaccines in a Mouse Mammary Cancer Model. BMC Immunol (2020) 21(1):25. 10.1186/s12865-020-00350-3 32370785 PMC7201681

[30] Mohsen MO Speiser DE Michaux J Pak H Stevenson BJ Vogel M Bedside Formulation of a Personalized Multi-Neoantigen Vaccine Against Mammary Carcinoma. J Immunother Cancer (2022) 10(1):e002927. 10.1136/jitc-2021-002927 35017147 PMC8753436

[31] Lin W Liu J Chen J Li J Qiu S Ma J Peptides of Tetraspanin Oncoprotein CD151 Trigger Active Immunity Against Primary Tumour and Experimental Lung Metastasis. EBioMedicine (2019) 49:133–44. 10.1016/j.ebiom.2019.10.025 31668880 PMC6945203

[32] Lhuillier C Rudqvist NP Yamazaki T Zhang T Charpentier M Galluzzi L Radiotherapy-Exposed CD8+ and CD4+ Neoantigens Enhance Tumor Control. J Clin Invest (2021) 131(5):e138740. 10.1172/JCI138740 33476307 PMC7919731

[33] Li L Zhang X Wang X Kim SW Herndon JM Becker-Hapak MK Optimized Polyepitope Neoantigen DNA Vaccines Elicit Neoantigen-Specific Immune Responses in Preclinical Models and in Clinical Translation. Genome Med (2021) 13(1):56. 10.1186/s13073-021-00872-4 33879241 PMC8059244

[34] Johnstone CN Smith YE Cao Y Burrows AD Cross RS Ling X Functional and Molecular Characterisation of EO771.LMB Tumours, a New C57BL/6-Mouse-Derived Model of Spontaneously Metastatic Mammary Cancer. Dis models Mech (2015) 8(3):237–51. 10.1242/dmm.017830 PMC4348562 25633981

[35] Brito Baleeiro R Liu P Chard Dunmall LS Di Gioia C Nagano A Cutmore L Personalized Neoantigen Viro-Immunotherapy Platform for Triple-Negative Breast Cancer. J Immunother Cancer (2023) 11(8):e007336. 10.1136/jitc-2023-007336 37586771 PMC10432671

[36] Zhang D Lin Z Wu M Cai Z Zheng Y He L Cytosolic Delivery of Thiolated Neoantigen Nano-Vaccine Combined With Immune Checkpoint Blockade to Boost Anti-Cancer T Cell Immunity. Adv Sci (2021) 8(6):2003504. 10.1002/advs.202003504 PMC7967047 33747739

[37] Yang SF Weng MT Liang JD Chiou LL Hsu YC Lee YT Neoantigen Vaccination Augments Antitumor Effects of Anti-PD-1 on Mouse Hepatocellular Carcinoma. Cancer Lett (2023) 563:216192. 10.1016/j.canlet.2023.216192 37088327

[38] Chen H Li Z Qiu L Dong X Chen G Shi Y Personalized Neoantigen Vaccine Combined With PD-1 Blockade Increases CD8+ Tissue-Resident Memory T-Cell Infiltration in Preclinical Hepatocellular Carcinoma Models. J Immunother Cancer (2022) 10(9):e004389. 10.1136/jitc-2021-004389 36113894 PMC9486396

[39] Zhao Q Wang Y Zhao B Chen H Cai Z Zheng Y Neoantigen Immunotherapeutic-Gel Combined With TIM-3 Blockade Effectively Restrains Orthotopic Hepatocellular Carcinoma Progression. Nano Lett (2022) 22(5):2048–58. 10.1021/acs.nanolett.1c04977 35133159

[40] Hos BJ Camps MGM van den Bulk J Tondini E van den Ende TC Ruano D Identification of a Neo-Epitope Dominating Endogenous CD8 T Cell Responses to MC-38 Colorectal Cancer. Oncoimmunology (2019) 9(1):1673125. 10.1080/2162402X.2019.1673125 32923109 PMC7458608

[41] Schrors B Hos BJ Yildiz IG Lower M Lang F Holtsträter C MC38 Colorectal Tumor Cell Lines From Two Different Sources Display Substantial Differences in Transcriptome, Mutanome and Neoantigen Expression. Front Immunol (2023) 14:1102282. 10.3389/fimmu.2023.1102282 36969213 PMC10030996

[42] Slansky JE Rattis FM Boyd LF Fahmy T Jaffee EM Schneck JP Enhanced Antigen-Specific Antitumor Immunity With Altered Peptide Ligands That Stabilize the MHC-Peptide-TCR Complex. Immunity (2000) 13(4):529–38. 10.1016/s1074-7613(00)00052-2 11070171

[43] Feola S Chiaro J Martins B Russo S Fusciello M Ylösmäki E A Novel Immunopeptidomic-Based Pipeline for the Generation of Personalized Oncolytic Cancer Vaccines. Elife (2022) 11:e71156. 10.7554/eLife.71156 35314027 PMC8989416

[44] Li M Bharadwaj U Zhang R Zhang S Mu H Fisher WE Mesothelin Is a Malignant Factor and Therapeutic Vaccine Target for Pancreatic Cancer. Mol Cancer Ther (2008) 7(2):286–96. 10.1158/1535-7163.MCT-07-0483 18281514 PMC2929838

[45] Leao IC Ganesan P Armstrong TD Jaffee EM. Effective Depletion of Regulatory T Cells Allows the Recruitment of Mesothelin-Specific CD8^+^ T Cells to the Antitumor Immune Response Against a Mesothelin-Expressing Mouse Pancreatic Adenocarcinoma. Clin Translational Sci (2008) 1(3):228–39. 10.1111/j.1752-8062.2008.00070.x PMC2847413 20357913

[46] Kinkead HL Hopkins A Lutz E Wu AA Yarchoan M Cruz K Combining STING-Based Neoantigen-Targeted Vaccine With Checkpoint Modulators Enhances Antitumor Immunity in Murine Pancreatic Cancer. JCI Insight (2018) 3(20):e122857. 10.1172/jci.insight.122857 30333318 PMC6237485

[47] Nagaoka K Sun C Kobayashi Y Kanaseki T Tokita S Komatsu T Identification of Neoantigens in Two Murine Gastric Cancer Cell Lines Leading to the Neoantigen-Based Immunotherapy. Cancers (Basel) (2021) 14(1):106. 10.3390/cancers14010106 35008270 PMC8750027

[48] Sun C Nagaoka K Kobayashi Y Maejima K Nakagawa H Nakajima J Immunotherapies Targeting Neoantigens Are Effective in PD-1 Blockade-Resistant Tumors. Int J Cancer (2023) 152(7):1463–75. 10.1002/ijc.34382 36451303

[49] Chen T Hu R Wan Y Sun F Wang Z Yue J Comprehensive Mutanome Analysis of Lewis Lung Cancer Reveals Immunogenic Neoantigens for Therapeutic Vaccines. Biochem Biophysical Res Commun (2020) 525(3):607–13. 10.1016/j.bbrc.2020.02.132 32115148

[50] Sun J Zhang J Hu H Qin H Liao X Wang F Anti-Tumour Effect of Neo-Antigen-Reactive T Cells Induced by RNA Mutanome Vaccine in Mouse Lung Cancer. J Cancer Res Clin Oncol (2021) 147(11):3255–68. 10.1007/s00432-021-03735-y 34291357 PMC8484245

[51] Sun C Nagaoka K Kobayashi Y Nakagawa H Kakimi K Nakajima J. Neoantigen Dendritic Cell Vaccination Combined With Anti-CD38 and CpG Elicits Anti-Tumor Immunity Against the Immune Checkpoint Therapy-Resistant Murine Lung Cancer Cell Line LLC1. Cancers (Basel) (2021) 13(21):5508. 10.3390/cancers13215508 34771674 PMC8583214

[52] Qin H Hu H Liao X Zhao P He W Su X Antitumor Effect of Neoantigen-Reactive T Cells Combined With PD1 Inhibitor Therapy in Mouse Lung Cancer. J Cancer Res Clin Oncol (2023) 149(10):7363–78. 10.1007/s00432-023-04683-5 36933035 PMC10024025

[53] Khurana D Martin EA Kasperbauer JL O'Malley BW Jr Salomao DR Chen L Characterization of a Spontaneously Arising Murine Squamous Cell Carcinoma (SCC VII) as a Prerequisite for Head and Neck Cancer Immunotherapy. Head Neck (2001) 23(10):899–906. 10.1002/hed.1130 11592238

[54] Dolina JS Lee J Brightman SE McArdle S Hall SM Thota RR Linked CD4+/CD8+ T Cell Neoantigen Vaccination Overcomes Immune Checkpoint Blockade Resistance and Enables Tumor Regression. J Clin Invest (2023) 133(17):e164258. 10.1172/JCI164258 37655661 PMC10471175

[55] Zolkind P Przybylski D Marjanovic N Nguyen L Lin T Johanns T Cancer Immunogenomic Approach to Neoantigen Discovery in a Checkpoint Blockade Responsive Murine Model of Oral Cavity Squamous Cell Carcinoma. Oncotarget (2017) 9(3):4109–19. 10.18632/oncotarget.23751 29423108 PMC5790525

[56] Liu CJ Schaettler M Blaha DT Bowman-Kirigin JA Kobayashi DK Livingstone AJ Treatment of an Aggressive Orthotopic Murine Glioblastoma Model With Combination Checkpoint Blockade and a Multivalent Neoantigen Vaccine. Neuro-Oncology (2020) 22(9):1276–88. 10.1093/neuonc/noaa050 32133512 PMC7523441

[57] Johanns TM Ward JP Miller CA Wilson C Kobayashi DK Bender D Endogenous Neoantigen-Specific CD8 T Cells Identified in Two Glioblastoma Models Using a Cancer Immunogenomics Approach. Cancer Immunol Res (2016) 4(12):1007–15. 10.1158/2326-6066.CIR-16-0156 27799140 PMC5215735

[58] Su T Liu X Lin S Cheng F Zhu G. Ionizable Polymeric Nanocarriers for the Codelivery of Bi-Adjuvant and Neoantigens in Combination Tumor Immunotherapy. Bioactive Mater (2023) 26:169–80. 10.1016/j.bioactmat.2023.02.016 PMC9982230 36883121

[59] Scheetz L Kadiyala P Sun X Son S Hassani Najafabadi A Aikins M Synthetic High-Density Lipoprotein Nanodiscs for Personalized Immunotherapy Against Gliomas. Clin Cancer Res (2020) 26(16):4369–80. 10.1158/1078-0432.CCR-20-0341 32439701 PMC7442596

[60] Swartz AM Congdon KL Nair SK Li QJ Herndon JE 2nd Suryadevara CM A Conjoined Universal Helper Epitope Can Unveil Antitumor Effects of a Neoantigen Vaccine Targeting an MHC Class I-Restricted Neoepitope. NPJ Vaccin (2021) 6(1):12. 10.1038/s41541-020-00273-5 PMC7814002 33462231

